# Learning restricted Boolean network model by time-series data

**DOI:** 10.1186/s13637-014-0010-5

**Published:** 2014-07-15

**Authors:** Hongjia Ouyang, Jie Fang, Liangzhong Shen, Edward R Dougherty, Wenbin Liu

**Affiliations:** 1 Department of Physics and Electronic Information Engineering, Wenzhou University, Wenzhou 325035, Zhejiang, China; 2 Department of Electrical and Computer Engineering, Texas A&M University, College Station 33101, TX, USA; 3 Computational Biology Division, Translational Genomics Research Institute, Phoenix 77843, AZ, USA

**Keywords:** Restricted Boolean network, Inference, Budding yeast cell cycle

## Abstract

Restricted Boolean networks are simplified Boolean networks that are required for either negative or positive regulations between genes. Higa et al. (BMC Proc 5:S5, 2011) proposed a three-rule algorithm to infer a restricted Boolean network from time-series data. However, the algorithm suffers from a major drawback, namely, it is very sensitive to noise. In this paper, we systematically analyze the regulatory relationships between genes based on the state switch of the target gene and propose an algorithm with which restricted Boolean networks may be inferred from time-series data. We compare the proposed algorithm with the three-rule algorithm and the best-fit algorithm based on both synthetic networks and a well-studied budding yeast cell cycle network. The performance of the algorithms is evaluated by three distance metrics: the normalized-edge Hamming distance $\mu_{\mathrm{ham}}^{e}$, the normalized Hamming distance of state transition $\mu_{\mathrm{ham}}^{\mathrm{st}}$, and the steady-state distribution distance *μ*^ssd^. Results show that the proposed algorithm outperforms the others according to both $\mu_{\mathrm{ham}}^{e}$ and $\mu_{\mathrm{ham}}^{\mathrm{st}}$, whereas its performance according to *μ*^ssd^ is intermediate between best-fit and the three-rule algorithms. Thus, our new algorithm is more appropriate for inferring interactions between genes from time-series data.

## 1 Introduction

A key goal in systems biology is to characterize the molecular mechanisms governing specific cellular behaviors and processes. This entails selecting a model class for representing the system structure and state dynamics, followed by the application of computational or statistical inference procedures to reveal the model structure from measurement data. The models of gene regulatory networks run the gamut from coarse-grained discrete networks to the detailed description of stochastic differential equations [[1]]. They provide a uniform way to study biological phenomena (e.g., cell cycle) and diseases (e.g., cancer) and ultimately lead to systems-based therapeutic strategies [[2]].

Boolean networks, and the more general class of probabilistic Boolean networks, are one of the most popular approaches for modeling gene networks. The inference of gene networks from high-throughput genomic data is an ill-posed problem. There exists more than one model that can explain the data. The search space for potential regulator sets and their corresponding Boolean functions generally increases exponentially with the number of genes in the network and the number of regulatory genes. It is particularly challenging in the face of small sample sizes, because the number of genes typically is much greater than the number of observations. Thus, estimates of modeling errors, which themselves are determined from the measurement data, can be highly variable and untrustworthy. Many inference algorithms have been proposed to elucidate the regulatory relationships between genes. Mutual information (MI) is an information-theoretic approach that can capture the nonlinear dependence between random variables. REVEAL is the first information-based algorithm to infer the regulatory relationships between genes [[3]]. However, a small MI does not necessarily mean that no regulatory relationship exists between genes (false negative). Conversely, a large MI does not necessarily mean a real regulatory relationship. ‘False-positive’ relationships often result from indirect interactions between two genes. The data processing inequality (DPI) and conditional mutual information (CMI) are two methods used to reduce the problem of false positives [[4],[5]]. Another information-based method is the minimum description length principle (MDL), which achieves a good trade-off between model complexity and fit to the data [[6]–[10]]. The coefficient of determination (CoD) selects a set of predictors whose expression levels can be used to better predict the expression of a target gene relative to the best possible prediction in the absence of observations [[11],[12]]. The best-fit extension incorporates inconsistencies generated from measurements or other unknown latent factors by constructing a network that makes as few misclassifications as possible [[13],[14]]. Any prior knowledge about the network structure or dynamics likely improves inference accuracy, especially for small sample sizes. Theoretical considerations and computational studies suggest that gene regulatory networks might operate close to a critical phase transition between ordered and disordered dynamical regimes [[15],[16]]. Liu et al. proposed a method to embed such a criticality assumption into the inference procedure. Such regularization of the sensitivity can both improve the inference and move the inferred networks closer to criticality [[17]].

A restricted Boolean network is a simplified Boolean model that has been used to study dynamical behavior of the yeast cell cycle [[18]–[24]]. In this model, the regulatory relationship between genes is either upregulation or downregulation. The output of the target gene is mainly dominated by the summation of its input genes. When the input summation is zero, the output state will remain as the current state of the target gene. The inference algorithm mentioned above generally cannot deal with this situation, and thus may not be appropriate to infer such network models. Recently, Higa et al. proposed a ‘three-rule algorithm’ to construct a restricted Boolean network from time-series data [[25]]. Their idea is that the consecutive state transitions of the system must be driven by some constraints, which can be induced from the small perturbations between two similar system states (detailed rules are provided in Section 3.1). However, the perturbations in microarry data sometimes may be caused by stochastic biological randomness or measurement process instead of real changes in gene expression level. This makes the three-rule algorithm inevitably lead to some incorrect constraints. In this paper, we propose a systematic method to infer a restricted Boolean network based on the state transitions of the target gene. Results of simulated networks and a modeled yeast cell cycle show that the proposed algorithm is more robust to noise than the three-rule method.

This paper is organized as follows: Background information and definitions are given in Section 2. Section 3 presents a brief introduction to the three rules; after which, we systematically analyze the regulatory relationships between input genes and their target gene and propose an inference algorithm. Section 4 and Section 5 present results for the simulated networks and for the cell cycle model of budding yeast. Concluding remarks are given in Section 6.

## 2 Background

### 2.1 Boolean networks

A Boolean network *G*(*V*, *F*) is defined by a set of nodes *V* = {*x*_1_, …, *x*_*n*_}, *x*_*i*_ ∈ {0, 1} and a set of Boolean functions *F* = {*f*_1_, …, *f*_*n*_} and $f_{i}:{\left\{0,1\right\}}^{k_{i}}\to \left\{0,1\right\}$. Each node *x*_*i*_ represents the expression state of gene *x*_*i*_, where *x*_*i*_ = 0 means that the gene is off, and *x*_*i*_ = 1 means it is on. Each node *x*_*i*_ is assigned a Boolean function $f_{i}\left(x_{1},\ldots ,x_{k_{i}}\right)$ with *k*_*i*_ specific input nodes, which is used to update its value. Under the synchronous updating scheme, all genes are updated simultaneously according to their corresponding update functions. The network's state at time *t* is represented by a binary vector *x*(*t*) = (*x*_1_(*t*), …, *x*_*n*_(*t*)). In the absence of noise, the state of the system at the next time step is

(1) $$x\left(t+1\right)=F\left(x_{1}\left(t\right),\ldots ,x_{n}\left(t\right)\right)$$

The long-run behavior of a deterministic Boolean network (BN) depends on the initial state, and the network will eventually settle down and cycle endlessly through a set of states called an attractor cycle. The set of all initial states that reach a particular attractor cycle forms the basin of attraction (BOA) for the cycle. Following a perturbation, the network in the long run may randomly escape an attractor cycle, be reinitialized, and then begin its transition process anew. For a BN with perturbation probability *p*, its corresponding Markov chain possesses a steady-state distribution. It has been hypothesized that attractors or steady-state distributions in Boolean formalisms correspond to different cell types of an organism or to cell fates. In other words, the phenotypic traits are encoded in the attractors [[1]]. There are two ways to define the perturbation probability *p*. One is that each gene can flip its state according to an i.i.d random perturbation vector *γ* = (*γ*_1_, ⋯, *γ*_*n*_), where *γ*_*i*_ ∈ {0, 1}, the *i*th gene flips if and only *γ*_*i*_ = 1, and *p* = *P*(*γ*_*i*_ = 1) for *i* = 1, 2, ⋯, *n*. The other is each state *x*(*t*) can transit to any other state with the same probability *p.* In this situation, at each time step, state *x*(*t*) will transit to the next state according to *F* with probability 1 + *p* − 2^*n*^ ∗ *p* and other states with probability *p*. In this paper, we adopt the later definition of the perturbation probability *p*.

### 2.2 Restricted Boolean networks

Restricted Boolean networks are simplified Boolean networks in which the regulatory relationships between genes obey the following convention: *a*_*ij*_ = 1 represents a positive regulation from gene *x*_*j*_ to *x*_*i*_ (activation); *a*_*ij*_ = − 1 represents a negative regulation from gene *x*_*j*_ to *x*_*i*_ (inhibition); and *a*_*ij*_ = 0 means that *x*_*j*_ has no effect on *x*_*i*_. The Boolean function $f_{i}\left(x_{1},\ldots ,x_{k_{i}}\right)$ is defined as [[18]]

(2) $$x_{i}\left(t+1\right)=\left\{\begin{array}{c} 1,\;\mathrm{if}\sum_{j\in \left\{1,\ldots ,k_{i}\right\}}a_{\mathrm{ij}}x_{i}\left(t\right)>0\\ 0,\;\mathrm{if}\sum_{j\in \left\{1,\ldots ,k_{i}\right\}}a_{\mathrm{ij}}x_{i}\left(t\right)<0\\ x_{i}\left(t\right),\;\mathrm{if}\sum_{j\in \left\{1,\ldots ,k_{i}\right\}}a_{\mathrm{ij}}x_{i}\left(t\right)=0. \end{array}\right)$$

This model is ‘restricted’ in the sense that functions satisfying formula (2) constitute a subset of the class of all Boolean functions. The number of restricted functions decreases dramatically as the input degree *k*_*i*_ increases. For example, there are 12 ($<2^{2^{2}}=16$) restricted functions for *k*_*i*_ = 2, and only 60 functions ($<<2^{2^{3}}=256$) for *k*_*i*_ = 3. The restricted model significantly reduces the model space, which is beneficial for inference, given a limited number of noisy high-throughput data.

## 3 Methods

### 3.1 Three-rule method

A time-series observation can be treated as a trajectory (or random walk) of the state space of the network used to model a real biological system. The three-rule method proposed by Higa et al. is to induce the constraints between genes from the small difference between two similar states and the difference between their next states [[25]]. Given an *m*-point time series S = {*S*(1), *S*(2), …, *S*(*m*)} of gene expression profiles, where *S*(*t*) ∈ {0, 1}^*n*^ for *t* = 1, 2, …, *m*, the three rules are as follows:

Rule 1: Let *S*(*t* − 1), *S*(*t*), and *S*(*t* + 1) be three consecutive states. If *S*(*t* − 1) and *S*(*t*) differ by a single gene *x*_*k*_, then for each gene *x*_*i*_ such that *x*_*i*_(*t*) ≠ *x*_*i*_(*t* + 1), we have *x*_*k*_ directly regulates *x*_*i*_; that is, *a*_*ik*_ ≠ 0.

Rule 2: Only the active genes at time *t* can possibly regulate genes at time *t* + 1.

Rule 3: Given two similar states *S*(*t*_1_) and *S*(*t*_2_), the difference between *S*(*t*_1_ + 1) and *S*(*t*_2_ + 1) must result from the genes in their predecessors *S*(*t*_1_) and *S*(*t*_2_) that are expressed differently.

Both rules 1 and 3 can also be extended to situations where *S*(*t* − 1) and *S*(*t*) or *S*(*t*_1_) and *S*(*t*_2_) differ in more than one gene. Cyclically applying these rules to any two states may lead to a group of constraint inequalities between variables *a*_*ij*_. Many available constraint satisfaction problem solvers (CSPs) [[26]] can be used to solve the possible regulatory relationships of one gene to the target gene.

**Figure 1:**
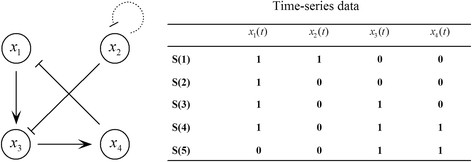
An example of four genes.

Rules 1 and 3 may give incorrect relationships if applied to noisy data; in other words, they are very sensitive to the noise inherent in data. We demonstrate this by using a small network that contains only four genes (see Figure 1). An arrow represents positive regulation, a line segment with a bar at the end represents negative regulation, and the dotted loop on *x*_2_ indicates that this gene downregulates itself. The time-series data at the right in Figure 1 are extracted from the network in Figure 1. Between *S*(1) and *S*(2), only *x*_2_ changes from 1 to 0, and only *x*_3_ flips from 0 to 1 in the successive states *S*(2) and *S*(3). We can conclude that *x*_2_ must inhibit *x*_3_ by applying rule 1, which means *a*_32_ = − 1 because turning off *x*_2_ turns on *x*_3_. If *S*(2) becomes 1001 owing to noise, then we will also have that gene *x*_4_ inhibiting *x*_2_, which means *a*_24_ = − 1.

### 3.2 Analysis of regulatory relationships based on constraints

In this section, we study the regulatory relationships based on the constraint inequalities in formula (2) and how the target gene switches from one state to another. The target gene can switch in one of four ways: 0 → 0, 0 → 1, 1 → 0, or 0 → 1. Given an input state, inactive genes have no effect on the target gene, which may help reduce the constraint inequalities of the summation ∑ _*j*_*a*_*ij*_*x*_*j*_(*t*) (1 ≤ *j* ≤ *k*_*i*_). Because the null input provides no constraints between *a*_*ij*_, we only need to investigate the non-null input situations.

First, consider the simplest situation where there is only one regulatory gene $x_{j_{1}}$. If gene $x_{j_{1}}$ is active and the target gene *x*_*i*_ switches from 0 to 1, then gene $x_{j_{1}}$ must activate the target gene *x*_*i*_ (which means $a_{ij_{1}}=1$). On the contrary, if the target gene *x*_*i*_ switches from 1 to 0, then it must be inhibited by $x_{j_{1}}$ (which means $a_{ij_{1}}=-1$). When the target gene *x*_*i*_ remains in state 1, we have $a_{ij_{1}}x_{j_{1}}\geq 0$ (which means $a_{ij_{1}}=1$). When the target gene *x*_*i*_ remains in state 0, we have $a_{ij_{1}}x_{j_{1}}\leq 0$ (which means $a_{ij_{1}}=-1$). We present the four possible regulatory relationships $a_{ij_{1}}$ in Table 1.

**Table 1:** Regulatory relationships for one input gene

**Number**	$x_{j_{1}}\left(t\right)$	***x***_***i***_**(*t*) →** ***x***_***i***_**(*t*** **+ 1)**	$a_{ij_{1}}$
1	1	0 → 0	−1
2	1	0 → 1	1
3	1	1 → 0	−1
4	1	1 → 1	1

When there are two regulatory genes $x_{j_{1}}$ and $x_{j_{2}}$, we only consider the input states 01, 10, and 11. If only one input gene is active, such as $x_{j_{1}}x_{j_{2}}=01$, then we can directly determine $a_{ij_{2}}$ from Table 1, whereas $a_{ij_{1}}$ remains totally non-determinant because it has no effect on the target gene. If both gene $x_{j_{1}}$ and gene $x_{j_{2}}$ are active, then we need to know whether or not the target gene *x*_*i*_ switches its state. First, if *x*_*i*_ switches from 1 to 0, then we have $a_{ij_{1}}=a_{ij_{2}}=-1$ to satisfy the constraint $a_{ij_{1}}+a_{ij_{2}}<0$. Similarly, if *x*_*i*_ switches from 0 to 1, then we have $a_{ij_{1}}=a_{ij_{2}}=1$ to satisfy the constraint $a_{ij_{1}}+a_{ij_{2}}>0$. Second, if *x*_*i*_ remains in state 0, then we have $a_{ij_{1}}=a_{ij_{2}}=-1$ or $a_{ij_{1}}=-a_{ij_{2}}$ because $a_{ij_{1}}+a_{ij_{2}}\leq 0$. Similarly, if *x*_*i*_ remains in state 1, then we have $a_{ij_{1}}=a_{ij_{2}}=1$ or $a_{ij_{1}}=-a_{ij_{2}}$ because $a_{ij_{1}}+a_{ij_{2}}\leq 0$. We call these later cases ‘semi-determined’ because there are two possible combinations of $a_{ij_{1}}$ and $a_{ij_{2}}$ in each case. In Table 2, we present the 12 possible regulatory relationships of $a_{ij_{1}}$ and $a_{ij_{2}}$ for two input genes.

**Table 2:** Regulatory relationships for two input genes

**Number**	$x_{j_{1}}\left(t\right)$	$x_{j_{2}}\left(t\right)$	***x***_***i***_**(*t*) →** ***x***_***i***_**(*t*** **+ 1)**	$a_{ij_{1}}$	$a_{ij_{2}}$	**Constraint**
1	0	1	0 → 0	No	−1	
2	1	0		−1	No	
3	1	1		−1 or 1	−1 or 1	$a_{ij_{1}}+a_{ij_{2}}\leq 0$
4	0	1	0 → 1	No	1	
5	1	0		1	No	
6	1	1		1	1	
7	0	1	1 → 0	No	−1	
8	1	0		−1	No	
9	1	1		−1	−1	
10	0	1	1 → 1	No	1	
11	1	0		1	No	
12	1	1		−1 or 1	−1 or 1	$a_{ij_{1}}+a_{ij_{2}}\geq 0$

No, totally undetermined; −1 or 1, semi-determined.

Analogously, the regulatory relationships for three input genes are shown in Table 3. There are 10 semi-determined cases, and most of them occur when the target gene *x*_*i*_ does not change. Some of the semi-determined cases in Tables 2 and 3 may become determined if some *a*_*ij*_ are determined. For example, given $a_{ij_{1}}+a_{ij_{2}}\leq 0$ for (3) in Table 2, we can determine $a_{ij_{2}}=1$ if $a_{ij_{1}}$ is determined to be 1. However, $a_{ij_{1}}$ still remains semi-determined (either 1 or −1) if $a_{ij_{1}}$ is determined to be −1. As the number of regulatory genes increases, the proportion of semi-determined cases increases significantly. We will not extend the above analysis to situations of more than three input genes. In most reference studies, the limit *k*_*i*_ ≤ 3 is generally respected to mitigate model complexity, particularly for small sample sizes.

**Table 3:** Regulatory relationships for three input genes

**Number**	$x_{j_{1}}\left(t\right)$	$x_{j_{2}}\left(t\right)$	$x_{j_{3}}\left(t\right)$	***x***_***i***_**(*t*) →** ***x***_***i***_**(*t*** **+ 1)**	$a_{ij_{1}}$	$a_{ij_{2}}$	$a_{ij_{3}}$	**Constraint**
1	0	0	1	0 → 0	No	No	−1	
2	0	1	0		No	−1	No	
3	1	0	0		−1	No	No	
4	0	1	1		No	−1 or 1	−1 or 1	$a_{ij_{2}}+a_{ij_{3}}\leq 0$
5	1	0	1		−1 or 1	No	−1 or 1	$a_{ij_{1}}+a_{ij_{3}}\leq 0$
6	1	1	0		−1 or 1	−1 or 1	No	$a_{ij_{1}}+a_{ij_{2}}\leq 0$
7	1	1	1		−1 or 1	−1 or 1	−1 or 1	$a_{ij_{1}}+a_{ij_{2}}+a_{ij_{3}}<0$
8	0	0	1	0 → 1	No	No	1	
9	0	1	0		No	1	No	
10	1	0	0		1	No	No	
11	0	1	1		No	1	1	
12	1	0	1		1	No	1	
13	1	1	0		1	1	No	
14	1	1	1		−1 or 1	−1 or 1	−1 or 1	$a_{ij_{1}}+a_{ij_{2}}+a_{ij_{3}}>0$
15	0	0	1	1 → 0	No	No	−1	
16	0	1	0		No	−1	No	
17	1	0	0		−1	No	No	
18	0	1	1		No	−1	−1	
19	1	0	1		−1	No	−1	
20	1	1	0		−1	−1	No	
21	1	1	1		−1 or 1	−1 or 1	−1 or 1	$a_{ij_{1}}+a_{ij_{2}}+a_{ij_{3}}<0$
22	0	0	1	1 → 1	No	No	1	
23	0	1	0		No	1	No	
24	1	0	0		1	No	No	
25	0	1	1		No	−1 or 1	−1 or 1	$a_{ij_{2}}+a_{ij_{3}}\geq 0$
26	1	0	1		−1 or 1	No	−1 or 1	$a_{ij_{1}}+a_{ij_{3}}\geq 0$
27	1	1	0		−1 or 1	−1 or 1	No	$a_{ij_{1}}+a_{ij_{2}}\geq 0$
28	1	1	1		−1 or 1	−1 or 1	−1 or 1	$a_{ij_{1}}+a_{ij_{2}}+a_{ij_{3}}>0$

No, totally undetermined; −1 or 1, semi-determined.

Given a target gene *x*_*i*_ and its predictor genes *x*_*j*_ (1 ≤ *j* ≤ *k*_*i*_), we may determine the value of *a*_*ij*_ at each time point *t* (1 ≤ *t* ≤ *m* − 1) by searching Tables 1, 2, or 3 across the whole time series S = {*S*(1), *S*(2), …, *S*(*m*)}. Let $N_{\mathrm{ij}}^{-1}$, $N_{\mathrm{ij}}^{1}$, and $N_{\mathrm{ij}}^{-1,1}$ denote the number of *a*_*ij*_ = − 1, *a*_*ij*_ = 1, and *a*_*ij*_ = − 1 or 1, respectively. The *degree of determination* of a regulatory relationship *a*_*ij*_ is defined as

(3) $$d_{\mathrm{ij}}=\left|N_{\mathrm{ij}}^{-1}-N_{\mathrm{ij}}^{1}\right|.$$

If $N_{\mathrm{ij}}^{-1}>N_{\mathrm{ij}}^{1}$, then *a*_*ij*_ is likely to be −1; otherwise, it is likely to be 1. The larger the value of *d*_*ij*_, the greater the determination of *a*_*ij*_. In order to reduce the semi-determined cases, we first find the one with the largest determination, say, *a*_*ij*,_, and determine its value by the majority rule. Then, we apply the value of *a*_*ij*_ to those inequalities including it to solve other semi-determined *a*_*ip*_ (*p* ≠ *j*, 1 ≤ *p*, *j* ≤ *k*_*i*_). By repeating this process, we can reduce the number of semi-determined cases and determine the values of other *a*_*ip*_ accordingly.

### 3.3 Error analysis

Given a predictor set for gene *x*_*i*_, the basic inconsistency is the discrepancy in the determination of *a*_*ij*_, and we define the error resulting from such an inconsistency by $\varepsilon_{{}_{\mathrm{ij}}}^{-1,1}=\min\left(N_{\mathrm{ij}}^{-1},N_{\mathrm{ij}}^{1}\right)$. A second kind of inconsistency arises from the null input. Specifically, the target gene *x*_*i*_ cannot flip its state under null input situations. Moreover, if it is negatively self-regulated (self-degradation), it cannot be active when its input genes are null. The number of such inconsistencies defines the error $\varepsilon_{{}_{i}}^{\mathrm{null}}$, which is listed in Table 4 for self-degradation and no self-degradation, respectively. The *total error* of a predictor set is defined by $\varepsilon =\varepsilon_{{}_{i}}^{\mathrm{null}}+\sum_{j}\varepsilon_{{}_{\mathrm{ij}}}^{-1,1}$. Generally, a consistent predicator set should have the minimal error and the minimal number of regulatory genes simultaneously.

**Table 4:** Errors in the null-input situations

**Number**	$x_{j_{1}}\left(t\right)=\cdots =x_{j_{\mathrm{ki}}}\left(t\right)$	***x***_***i***_**(*t*) →** ***x***_***i***_**(*t*** **+ 1)**	$\varepsilon_{i}^{\mathrm{null}}$	
			**Self-degradation regulated**	**No self-degradation**
1	0	0 → 0	0	0
2	0	0 → 1	1	1
3	0	1 → 0	0	1
4	0	1 → 1	1	0

### 3.4 A small example

We now apply the above analysis to infer the predicator set for gene *x*_3_ in Figure 1. Based on Tables 1,2,3,4, the results for all possible one- and two-input genes at each time point are presented in Tables 5,6,7,8, respectively. In those six possible predictor sets, the minimal error is achieved by *x*_1_ and *x*_2_, which are just the regulatory genes of *x*_3_.

**Table 5:** **Regulatory relationships** ***a***_**3** ***j***_**for one input** ***x***_**1**_**(or** ***x***_**2**_**or** ***x***_**4**_**) at each time step**

***t***	***x***_**1**_**(*t*)**	***x***_**2**_**(*t*)**	***x***_**4**_**(*t*)**	***x***_**3**_**(*t*) →** ***x***_**3**_**(*t*** **+ 1)**	***a***_**31**_	$\varepsilon_{3}^{\mathrm{null}}$	***a***_**32**_	$\varepsilon_{3}^{\mathrm{null}}$	***a***_**34**_	$\varepsilon_{3}^{\mathrm{null}}$
1	1	1	0	0 → 0	−1	0	−1	0		0
2	1	0	0	0 → 1	1	0		1		1
3	1	0	0	1 → 1	1	0		0		1
4	1	0	1	1 → 1	1	0		0	1	0

**Table 6:** **Regulatory relationships** ***a***_**3** ***j***_**for two inputs** ***x***_**1**_**and** ***x***_**2**_**at each time step**

***t***	***x***_**1**_**(*t*)**	***x***_**2**_**(*t*)**	***x***_**3**_**(*t*) →** ***x***_**3**_**(*t*** **+ 1)**	***a***_**31**_	***a***_**32**_	**Constraint**	$\varepsilon_{3}^{\mathrm{null}}$
1	1	1	0 → 0	−1,1	*−1*,1	*a*_31_ + *a*_32_ ≤ 0	0
2	1	0	0 → 1	1	No		0
3	1	0	1 → 1	1	No		0
4	1	0	1 → 1	1	No		0

The italicized value is solved from the determination *a*_31_ = 1.

**Table 7:** **Regulatory relationships** ***a***_**3** ***j***_**for two inputs** ***x***_**1**_**and** ***x***_**4**_**at each time step**

***t***	***x***_**1**_**(*t*)**	***x***_**4**_**(*t*)**	***x***_**3**_**(*t*) →** ***x***_**3**_**(*t*** **+ 1)**	***a***_**31**_	***a***_**34**_	**Constraint**	$\varepsilon_{3}^{\mathrm{null}}$
1	1	0	0 → 0	−1	No		0
2	1	0	0 → 1	1	No		0
3	1	0	1 → 1	1	No		0
4	1	1	1 → 1	−1,1	−1,1	*a*_31_ + *a*_34_ ≥ 0	0

**Table 8:** **Regulatory relationships** ***a***_**3** ***j***_**for two inputs** ***x***_**2**_**and** ***x***_**4**_**at each time step**

***t***	***x***_**2**_**(*t*)**	***x***_**4**_**(*t*)**	***x***_**3**_**(*t*) →** ***x***_**3**_**(*t*** **+ 1)**	***a***_**32**_	***a***_**34**_	**Constraint**	$\varepsilon_{3}^{\mathrm{null}}$
1	1	0	0 → 0	−1	No		0
2	0	0	0 → 1				1
3	0	0	1 → 1				0
4	0	1	1 → 1	No	1		0

### 3.5 Inference algorithm

Given a time series S = {*S*(1), *S*(2), …, *S*(*m*)}, the minimal error predictor sets may not be unique. Each of them can be viewed as fitting the target gene in a different way. We employ the heuristic that if one gene occurs frequently in those sets, then it is highly probably to be a true regulatory gene. Combining them may give a more reliable prediction and can also help alleviate the constraint of using at most three input genes for a target gene. Given a target gene *x*_*i*_, we propose the following algorithm to infer its regulatory gene set:

1. Calculate the total error of each combination of one, two, or three regulatory gene sets *P*(*x*_*i*_).

2. Sort the predictor sets in ascending order of their errors.

3. If a gene appears in the first *l* sets with a frequency greater than or equal to 50%, then it is selected as a regulatory gene.

## 4 Implementation

As mentioned in the introduction, many algorithms have been proposed to infer gene regulatory networks. A recent study shows that the best-fit algorithm appears to give the best results for the recovery of regulatory relationships among REVEAL, BIC, MDL, uMDL, and Best-Fit [[27]]. In this paper, we compare the performance of the three-rule algorithm, the best-fit algorithm and the proposed algorithm based on both synthetic networks as well as on a well-studied budding yeast cell cycle network.

We have implemented the three-rule algorithm and our proposed algorithm based on the PBN Toolbox (http://code.google.com/p/pbn-matlab-toolbox/), which includes the implementation of best-fit algorithm and the calculation of the steady state distribution and other intervention modules for Boolean networks. Genetic regulatory networks are commonly believed to have sparse connectivity topology. To evaluate the inference algorithms based on simulated time series of network states, we have restricted the random BNs to resemble this property of biological networks. Specifically, we have generated random BNs with a scale-free topology, and each gene has at most five predictors: $=\mathrm{ma}x_{i=1}^{n}k_{i}\leq 5$. We uniformly assign each gene 1 to *K* regulators that upregulate (1) or downregulate (−1) it. The average connectivity of random networks is (1 + *K*)/2.

In order to compare the performance of the three algorithms with the ground-truth network, we use the following three distances [[28],[29]]:

(1) The normalized-edge Hamming distance,

(4) $$\mu_{\mathrm{ham}}^{e}=\frac{\mathrm{FN}+\mathrm{FP}}{P+N},$$

where FN and FP represent the number of false-negative and false-positive wires, respectively. *P* and *N* represent the total number of positive and negative wires, respectively. This Hamming distance reflects the accuracy of the recovered regulatory relationships.

(2) The normalized Hamming distance of state transitions,

(5) $$\mu_{\mathrm{ham}}^{\mathrm{st}}=\frac{1}{n*2^{n}}\sum_{i=1}^{n}\sum_{k=1}^{2^{n}}\left[f_{i}\left(x_{k}\right)⊕f_{i}^{'}\left(x_{k}\right)\right],$$

where *f*_*i*_(•) and $f_{i}^{'}\left(•\right)$ represent the Boolean function of gene *i* in the ground-truth network and the inferred network, respectively; x_k_ represents a binary state vector, and ⊕ denotes modulo-2 addition. This Hamming distance indicates the accuracy of the inferred network for predicting the next state of the ground-truth network.

(3) The steady-state distribution distance,

(6) $$\mu^{\mathrm{ssd}}=\sum_{k=1}^{2^{n}}\left|\pi_{k}-\pi_{k}^{'}\right|,$$

where *π*_*k*_ and $\pi_{k}^{'}$ are the steady-state distribution of state *x*_*k*_ in the ground-truth network and the inferred network, respectively. The steady-state distribution distance reflects the degree of an inferred network approaching the long-run behavior of the ground-truth network.

## 5 Results and discussion

### 5.1 Simulated results

Owing to the computational complexity and the network state space, which increases exponentially with the number of genes or the network size, all our simulations are based on networks with *n* = 10 genes. We generate 300 random Boolean networks respectively with maximal input degree *K* = 3 and *K* = 5. For each simulated network, we generate about 4 time series so that the total time points add up to 40. Given a specific sample data, the noise is added by flipping the value of each bit with probability 0.05 and 0.10, respectively. The steady-state distribution is calculated by a perturbation parameter *p* = 0. 0001. For the proposed algorithm, we selected the first *l* = 10 minimal error predictor sets. For best fit, we selected the minimal error predictor sets from *k* = 1, 2, 3. In Table 9, we list the average number of true-positive and false-positive connections for *K* = 3 and *K* = 5 in different noise intensities.

**Table 9:** Average number of true-positive and false-positive connections for three algorithms

***K***	**Noise (%)**	**Algorithm**	***m*** **= 10**		***m*** **= 20**		***m*** **= 30**		***m*** **= 40**	
			**TP**	**FP**	**TP**	**FP**	**TP**	**FP**	**TP**	**FP**
3	0	Three-rule	6.2	0	8.7	0.6	11.3	1.6	13.3	3.0
		New	8.7	3.1	10.5	3.1	11.8	3.3	12.5	3.3
		Best-fit	8.1	4.6	10.2	5.4	12.2	6.4	13.3	7.0
	5	Three-rule	2.6	2.7	7.3	11.5	10.6	20.7	12.5	30.3
		New	7.0	7.5	8.7	6.9	10.1	6.3	10.7	6.3
		Best-fit	7.1	11.1	9.2	15.1	10.8	15.7	11.6	15.9
	10	Three-rule	1.8	3.6	6.5	17.6	10.5	31.6	12.4	39.8
		New	5.5	10.0	6.9	9.5	8.1	9.2	8.4	9.1
		Best-fit	6.0	15.2	8.1	19.1	9.2	19.3	9.9	19.0
5	0	Three-rule	6.7	0.1	8.9	0.6	11.0	1.3	12.6	2.3
		New	8.3	2.7	9.9	3.0	10.9	3.4	11.4	3.9
		Best-fit	8.2	4.6	10.1	5.4	11.8	6.4	12.7	6.9
	5	Three-rule	3.0	3.2	7.86	11.8	10.7	20.5	12.8	28.6
		New	6.7	7.6	8.4	7.0	9.3	6.7	9.8	6.3
		Best-fit	7.1	11.5	9.2	15.4	10.4	15.7	11.1	16.1
	10	Three-rule	2.7	2.8	6.9	16.5	10.6	31.6	12.4	39.4
		New	5.3	9.9	7.0	9.5	7.5	9.3	8.1	9.1
		Best-fit	7.2	11.5	8.2	18.9	9.0	19.3	9.4	19.4

**Figure 2:**
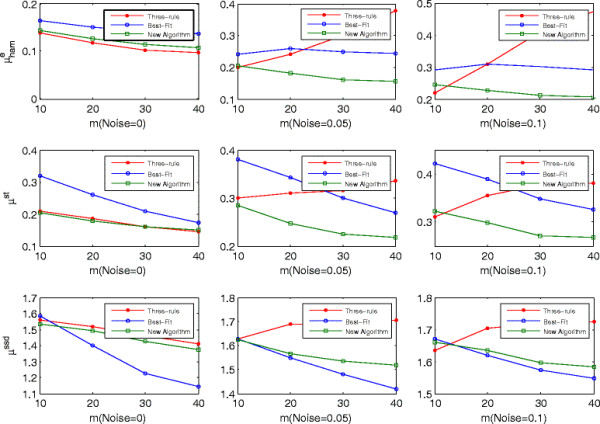
**Comparison of**$\mu_{\mathrm{ham}}^{e}$**,**$\mu_{\mathrm{ham}}^{\mathrm{st}}$**,** ***μ***^**ssd**^**for the three algorithms with 0%, 5%, and 10%noises (*K*** **= 3).**

Figure 2 shows the performance of three algorithms on networks with *K* = 3 under different noise intensities according to three distance metrics: the normalized-edge Hamming distance $\mu_{\mathrm{ham}}^{e}$, the normalized Hamming distance of state transition $\mu_{\mathrm{ham}}^{\mathrm{st}}$, and the steady-state distribution distance *μ*^ssd^. The performance of the three-rule algorithm and the proposed algorithm is very close when there is no noise. However, it differs dramatically in noisy data. Specifically, the performance of the proposed algorithm increases as the sample size increases while that of the three-rule algorithm decreases. The main reason lies in the fact that the proposed algorithm infers the regulatory relation based on the entire time series instead of on a small perturbation between two time points, which makes it more robust against noise than the three-rule algorithm. Given a specific noise intensity *η*, with more samples, there are more noisy perturbed bits; so, more incorrect connections will be inferred by the three-rule algorithm. Table 9 shows that the number of the false positives of the three-rule algorithm increases more quickly than that of the true positives as the sample size increases. This is the main factor which makes its performance deteriorate even though the sample size increases. Consequently, the three-rule algorithm is very sensitive to noise in the data, and increasing sample size makes no improvement in its performance.

**Figure 3:**
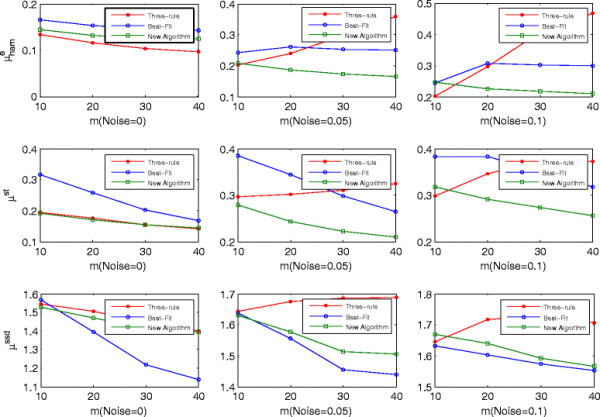
**Comparison of**$\mu_{\mathrm{ham}}^{e}$**,**$\mu_{\mathrm{ham}}^{\mathrm{st}}$**,** ***μ***^**ssd**^**for the three algorithms with 0%, 5%, and 10%noises (*K*** **= 5).**

Compared with the best-fit algorithm, the proposed algorithm performs better with respect to $\mu_{\mathrm{ham}}^{e}$ and $\mu_{\mathrm{ham}}^{\mathrm{st}}$. In a restricted Boolean network model, the output of states with $\sum_{j}a_{\mathrm{ij}}x_{i}\left(t\right)=0$ is determined by the current state of the target gene *x*_*i*_. This means that given the same input state, *x*_*i*_ may be 1 at one time and be 0 at another time. The best-fit algorithm does not allow such situation, and it will treat such a case in the data as an error. If the target gene *x*_*i*_ has three regulators and one downregulates it, then there will be 3 such states out of the 8 possible input states. The influence of such cases on the performance of best-fit algorithm can not be neglected. Additionally, the best-fit algorithm cannot deal with the inconsistency listed in Figure 3. These two factors hurt its performances as compared to the proposed algorithm on $\mu_{\mathrm{ham}}^{e}$ and $\mu_{\mathrm{ham}}^{\mathrm{st}}$. Table 9 shows that the number of the true positives of both algorithms is almost the same, but the number of false positives of the best-fit algorithm is larger than that of the proposed algorithm.

Concerning the steady state distribution distance *μ*^ssd^, the proposed algorithm performs not so well as the best-fit algorithm. However, their difference decreases as the noise intensity increases. As pointed in [[27]], the inferred networks with relative more connections can explain the observed data better with respect to steady-state distribution distance *μ*^ssd^, even though some are incorrect connections. Because the best-fit algorithm infers more connection than the proposed algorithm (see Table 9), it performs better on *μ*^ssd^ than the latter. On the other hand, the proposed algorithm is more robust than the best-fit algorithm as it combines those minimal error sets to determine the regulatory gene instead of selecting one. When noise intensity increases, the performance of the best-fit algorithm will drop more quickly than that of the proposed algorithm, which leads to their performance on *μ*^ssd^ converges.

**Figure 4:**
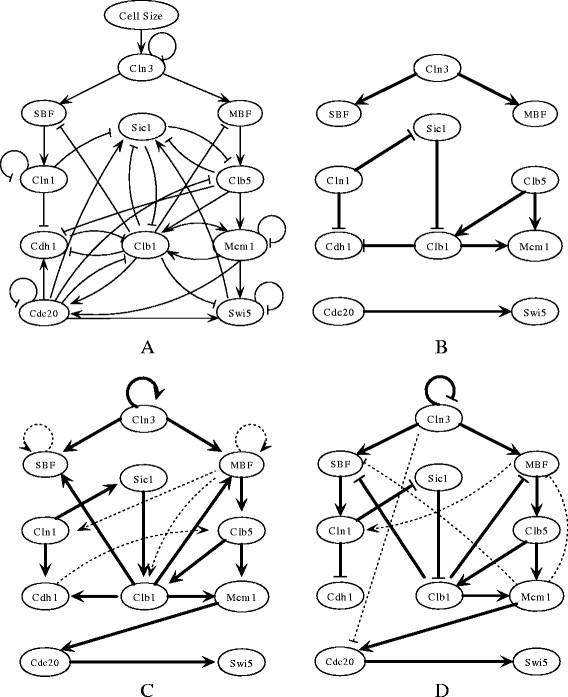
**The original and inferred cell cycle networks of budding yeast. (A)** Original network. **(B)** Network inferred by three-rule method. **(C)** Network inferred by the best-fit algorithm. **(D)** Network inferred by the proposed algorithm. In **(A)**, **(B)**, and **(D)**, arrows denote positive regulation; ‘T’ lines are negative regulation; and ‘T loops are self-degradation. In **(B)**, **(C)**, and **(D)**, bold solid lines denote the correct inferred regulatory relationships, and the light dashed lines denote the incorrectly inferred regulatory relationships.

Figure 4 shows the performance of three algorithms on networks with *K* = 5, which are analogous to the trends observed in Figure 2. The only difference is that the performance of the three algorithms decreases because the networks' complexity makes them hard to infer. In summary, the proposed algorithm performs better than the three-rule algorithm on the three distance metrics in noisy situations, whereas it performs less well than the best-fit algorithm on the steady-state distribution distance. This suggests that it is more feasible to infer the structure of restricted Boolean network model than the three-rule algorithm and best-fit algorithm.

### 5.2 Cell cycle model of budding yeast

The cell cycle is a vital biological process in which one cell grows and divides into two daughter cells. It consists of four phases, G1, S, G2, and M, and is regulated by a highly complex network that is highly conserved among the eukaryotes. From the 800 genes involved in the cell cycle process of budding yeast, Li et al. constructed a network of 11 key regulators: Cln3, MBF, SBF, Cln1, Cdh1, Swi5, Cdc20, Clb5, Sic1, Clb1, and Mcm1 [[18]]. This restricted Boolean network model (shown in Figure 4A) has an attractor whose biggest basin corresponds to the biological G1 stationary state. The temporal sequence in Table 10 is a pathway from this basin, which follows the biological trajectory of the cell cycle network.

**Table 10:** Temporal evolution of state for cell cycle

**Time**	**Cln3**	**MBF**	**SBF**	**Cln1**	**Cdh1**	**Swi5**	**Cdc20**	**Clb5**	**Sic1**	**Clb1**	**Mcm1**	**Phase**
1	1	0	0	0	1	0	0	0	1	0	0	Start
2	0	1	1	0	1	0	0	0	1	0	0	G1
3	0	1	1	1	0	0	0	0	1	0	0	G1
4	0	1	1	1	0	0	0	0	0	0	0	G1
5	0	1	1	1	0	0	0	1	0	0	0	S
6	0	1	1	1	0	0	0	1	0	1	1	G2
7	0	0	0	1	0	0	1	1	0	1	1	M
8	0	0	0	0	0	1	1	0	0	1	1	M
9	0	0	0	0	0	1	1	0	1	1	1	M
10	0	0	0	0	0	1	1	0	1	0	1	M
11	0	0	0	0	1	1	1	0	1	0	0	M
12	0	0	0	0	1	1	0	0	1	0	0	M
13	0	0	0	0	1	0	0	0	1	0	0	G1

We have applied the three algorithms to the above artificial time-series data and show the inferred networks in Figure 4. In the simplified model of the budding yeast cell cycle, there are a total of 34 regulatory relationships (or connections). The three-rule algorithm inferred 10 relationships, all correct (see Figure 4B). The best-fit algorithm inferred 15 correct and 5 incorrect relationships (see Figure 4C). The proposed algorithm inferred 15 correct and 4 incorrect relationships (see Figure 4D). Both best-fit and the proposed algorithms inferred more true regulatory relationships than the three-rule algorithm with some incorrect connections. For studying regulatory relationships, this may be more advantageous because more potential regulatory relationships are made available for biologists to check in the wet lab.

We also ran 100 simulations with 5% and 10% noises for this pathway. Even for the same pathway data, the result of each noisy pathway data differs dramatically. This is not surprising because noise significantly influences the determination of regulatory relations for all algorithms. The performance of the three algorithms on $\mu_{\mathrm{ham}}^{e}$, $\mu_{\mathrm{ham}}^{\mathrm{st}}$, and *μ*^ssd^ is listed Table 11. The relative performance of the three algorithms for this pathway data is also consistent with the previous simulation results.

**Table 11:** The performance of the three algorithms for the yeast-pathway data

	**Noise**								
	**0**%			**5**%			**10**%		
	**Distance**								
	$\mu_{\mathrm{ham}}^{e}$	$\mu_{\mathrm{ham}}^{\mathrm{st}}$	***μ***^**ssd**^	$\mu_{\mathrm{ham}}^{e}$	$\mu_{\mathrm{ham}}^{\mathrm{st}}$	***μ***^**ssd**^	$\mu_{\mathrm{ham}}^{e}$	$\mu_{\mathrm{ham}}^{\mathrm{st}}$	***μ***^**ssd**^
Three-rule	0.198	0.313	1.394	0.27	0.378	1.454	0.29	0.402	1.472
New algorithm	0.19	0.250	1.372	0.252	0.304	1.386	0.292	0.334	1.438
Best-fit	0.198	0.229	1.245	0.298	0.341	1.263	0.365	0.403	1.298

### 5.3 Computational issues

When inferring real networks with moderate size, the time complexity of algorithms is a key issue. Almost all algorithms proposed to date possess exponential complexity. The time complexity of the proposed algorithm and best-fit algorithm is $\left(n\cdot C_{n}^{k}\cdot m\right)$. The most time-consuming process for the three-rule algorithm is to solve the constraint inequalities, and its time complexity is *O*(*n* ⋅ *c*^*n*^ ⋅ *m*^2^) (1 < *c* < 2). From this point of view, the three-rule algorithm is more time consuming than the other two.

The proposed algorithm is similar in workflow to the best-fit algorithm; however, additional computation time results from three factors: (1) determination of the possible regulatory relationships, (2) determination during error estimation if an output state is correct for a given model according to Equation (2), and (3) combination of the first ten least-error models in the last step.

In practice, however, algorithm complexity is not the limiting factor. As shown in Table 12, for 11, 12, and 13 genes, and for *N* = 20 and *N* = 40, the proposed algorithm's computation time is between the best-fit and the three-rule algorithms, but the overriding computational issue is computation of the steady-state distribution, which is often required for application. It is for this reason that interest has focused on reducing network complexity [[29]–[31]].

**Table 12:** Algorithm timings (seconds)

***n***	***N*** **= 20**			***N*** **= 40**			**SSD**
	**Three-rule**	**Best-fit**	**Proposed**	**Three-rule**	**Best-fit**	**Proposed**	
11	1.04	0.09	1.11	2.7	0.14	1.67	25
12	2.5	0.11	2.63	4.1	0.18	2.15	160
13	6.3	0.15	3.55	7.5	0.23	4.11	1,500

## 6 Conclusion

The model space of Boolean networks is huge and from the point of view of evolution, it is unimaginable for nature to select its operational mechanisms from such a large space. Restricted Boolean networks, as a simplified model, have recently been extensively used to study the dynamical behavior of the yeast cell cycle process. In this paper, we propose a systematic method to infer the restricted Boolean network from time-series data. We compare the performance of the three-rule, best-fit, and the proposed algorithms both on simulated networks and on an artificial model of budding yeast. Results show that our algorithm performs better than the three-rule and best-fit algorithms according to the distance metrics $\mu_{\mathrm{ham}}^{e}$ and $\mu_{\mathrm{ham}}^{\mathrm{st}}$, but slightly less well than the best-fit algorithm according to *μ*^ssd^. This result indicates that the proposed algorithm may be more appropriate for recovering regulatory relationships between genes under the restricted Boolean network model.

The main advantage of the proposed algorithm is that it is more robust to noise than both the three-rule algorithm and best-fit algorithm. The proposed algorithm infers the regulatory relationships according to the consecutive state transitions of the target gene, instead of the small perturbations between two similar states in the three-rule algorithm. Simulation results show that noise in the data may induce many incorrect constraints by the three-rule algorithm. This hinders its application to noisy samples. Moreover, the proposed algorithm can capture the intrinsic state transition defined in Equation 2, whereas the best-fit algorithm cannot. Hence, because the inference processes of both algorithms try to find the minimal-error predictor set, the proposed algorithm can distinguish error in the data more accurately than the best-fit algorithm. Additionally, combination of the minimal error predictor sets in the proposed algorithm also improves its robustness.

In the Boolean formalism, a single time series (or trajectory) can be treated as a random walk across state space. It is not possible to recover the complex biological system from just one short trajectory by any method. Using heterogeneous data and some *a priori* knowledge is typically a necessity. *A priori* knowledge can be incorporated into the proposed algorithm and helps by reducing the search space. For instance, an algorithm might assume a prescribed attractor structure [[32]]. In our case, if we know that *x* regulates *y*, then we only consider those combinations containing *x*, thereby reducing the search space. Additionally, different methods may focus on different aspects of the inference process. For example, the best-fit algorithm and CoD are mainly concerned with the fitness of the data, whereas MDL-based methods intend to reduce structural risks. Future work will involve combining MDL with the proposed algorithm to reduce the rate of false positives.

## Competing interests

The authors declare that they have no competing interests.
